# Video livestreaming in emergency trauma dispatch: an observational study of technological integration with clinical decision-making in prehospital enhanced care services

**DOI:** 10.1186/s13049-025-01406-2

**Published:** 2025-06-19

**Authors:** Scott Munro, Lucie Ollis, Carin Magnusson, Jill Maben, Cath Taylor

**Affiliations:** 1 School of Health Sciences, University of Surrey, Guildford, Surrey UK; 2 South East Coast Ambulance Service NHS Foundation Trust, Crawley, West Sussex UK

**Keywords:** Video, Smartphone, Emergency medical services, Prehospital, Decision-making, Trauma, Situational awareness, Qualitative research, Ethnography, Interview

## Abstract

**Background:**

Emergency Medical Services (EMS) play a critical role as the initial point of contact for patients with trauma injuries, where making timely and accurate dispatch decisions is crucial for determining the speed and effectiveness of the response. Assessing injury severity and the appropriate EMS resources needed based on audio medical emergency number calls (e.g. 999/911/112) alone presents challenges. The prevalence of smartphones among the UK population offers a unique opportunity by enabling callers to send live video feeds to Emergency Operations Centres. This study explores the use of video livestreaming in emergency dispatch of prehospital enhanced care teams to determine how and why it impacts decision-making and situational awareness during trauma incidents and whether this varies by patient/caller, incident or dispatcher characteristics.

**Methods:**

A multimethod qualitative observational study was undertaken comprising 200 h of non-participant ethnographic observation of the use of video livestreaming in routine practice, and 14 semi-structured interviews with staff within two critical care services in London, UK who used the technology. Data collection and analysis were underpinned by naturalistic decision-making models that emphasise the role of situational awareness. Data were analysed and triangulated using the framework method.

**Findings:**

We identified three phases in the decision-making process for use of video livestreaming in emergency dispatch: (i) Evaluation and Determination, (ii) Integration and Observation, and (iii) Resolution and Response. Phase 1 addresses why video livestreaming is used and the patient/caller, incident and dispatcher characteristics and identifying primary drivers and barriers. Phase 2 explores how livestreaming impacts situational awareness, focusing on visual cues such as clinical indicators, mechanisms of injury, and environmental factors. Phase 3 examines the impact on dispatch decision-making and immediate care advice. An overarching theme emphasises the role of dispatchers' clinical experience and expertise in using video livestreaming effectively.

**Conclusions:**

Video livestreaming has the potential to impact situational awareness and decision-making in emergency dispatch, as reported by participants and observed during the study, particularly in response to complex and ambiguous trauma scenarios. The technology's effectiveness depends on dispatcher expertise, caller characteristics, and incident complexity. Further research is needed to evaluate its use across different EMS contexts.

**Supplementary Information:**

The online version contains supplementary material available at 10.1186/s13049-025-01406-2.

## Introduction

### Background and rationale

Traumatic injuries present a serious public health challenge and are responsible for high mortality and morbidity worldwide [1]. Emergency Medical Services (EMS) are the frontline in trauma response, serving as the first medical point of contact for patients with time-critical and/or traumatic injuries. When EMS suspects a severe injury, such as major trauma, specialist paramedics in critical care and/or helicopter EMS (HEMS) may be dispatched to the scene to provide care for the patient and/or transport them to the hospital [2]. The significance of decision-making and accurate dispatch decisions in these scenarios is crucial, as it impacts on the speed and type of the EMS response provided to patients [3]. Understanding the severity of injuries and the necessary EMS resources required from audio emergency medical calls alone remains a challenge due to the inherent subjectivity of descriptions, the emotional stress of lay public 999 callers, and their limited medical knowledge or training, and occasionally language barriers [2–5].

The widespread ownership of smartphones in the UK general population (90%) [6] has provided a potential opportunity to address some of these challenges in the EMS setting [4, 7, 8]. Smartphone technology allows emergency medical callers to stream real-time live video feeds from emergency scenes directly to the Emergency Operations Centre, where emergency medical calls are received and decisions regarding resource deployment are made [4, 7, 9].

The observational study reported here was nested within a larger feasibility randomised controlled trial investigating the use of video livestreaming for trauma incidents [10]. This study was conducted in one HEMS and one land-based prehospital critical care service in London, UK, both routinely using video livestreaming, to answer the following question: How and why does the use of video livestreaming in emergency dispatch of prehospital enhanced care teams impact decision-making and situational awareness during trauma incidents, and does this impact vary by patient/caller characteristics, incident specifics, or dispatcher attributes?

## Methods

This study employed qualitative methods, including semi-structured interviews and non-participant ethnographic observations, to explore the use of video livestreaming in dispatch decision-making during trauma incidents. The COREQ checklist guided data collection and study reporting [11].

### Setting

Data for this study were gathered from two specialist prehospital Enhanced Care Teams (ECTs) in London: the London Air Ambulance (LAA) (a physician led HEMS) and the Advanced Paramedic Practitioners in Critical Care team (APP-CC) from the London Ambulance Service NHS Trust (LAS). In the year 2023–24, the LAS received 1,922,066 emergency calls [12], making it the busiest ambulance service in the UK. The service covers an area of 620 square miles and serves nearly 9 million residents in London [13].

In the LAS, emergency medical calls are initially answered and triaged by non-clinically trained call handlers using the Medical Priority Dispatch System [14]. This process generates incidents on the Computer Aided Dispatch system, which are then queued for assignment by non-clinically trained ambulance dispatchers.

In addition to the frontline ambulance crews, ECTs such as the LAA and APP-CC provide specialist skills beyond those of standard ambulance crews. These teams are specially dispatched by clinical members working in the LAS Emergency Operations Centre, such as HEMS paramedics and APP-CCs, who screen incidents (by reading from data entered by the call takers and/or listening to the calls) for specific words or terms believed to indicate critically injured patients, to determine if their specialist resources are needed. Throughout this manuscript, the term *clinician dispatcher* will refer to team members responsible for dispatching APP-CCs and HEMS teams, emphasising their dual role in resource allocation and applying clinical expertise to assess and assign tasks.

Since 2019, both the LAA and APP-CC have incorporated the'GoodSAM instant-on-scene' [15] software for video livestreaming into routine practice. The clinician dispatcher sends the 999 caller a text message with a link to request consent to access to their phone’s location and camera. Once consent is provided, the phone streams live video directly to the Emergency Operations Centre. The footage is not recorded or stored.

### Theoretical framework

**Fig. 1:**
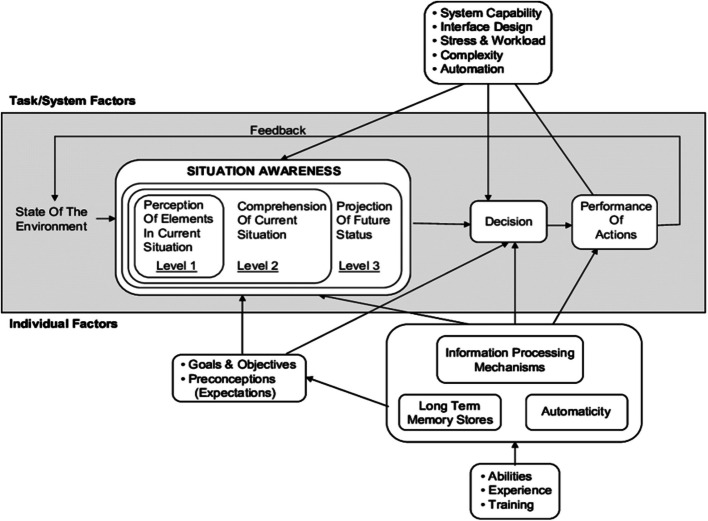
Endsley’s Model of situation awareness in dynamic decision making [17]. (Reprinted by Permission of SAGE Publications)

Data collection and analysis drew on Naturalistic Decision-Making (NDM) theories [16]. This included Endsley’s model of situational awareness (SA) [17], containing three levels of awareness: perception of elements in the current situation, comprehension of their meaning, and projection of future status (Fig. 1). SA is depicted as the operator's internal model of the state of the environment and is the main precursor to decision-making [18].

### Research team

A qualitative subgroup from the SEE-IT Trial [10], comprising the authors SM, CT, JM, LO, and CM undertook this study. The primary researcher undertaking observation shifts and interviews (SM) was a male research paramedic and specialist paramedic in critical care employed by the South East Coast Ambulance Service NHS Foundation Trust at the time of the study, SM has prior experience conducting mixed methods research. No prior relationships existed between the research team and any of the study participants. To account for the influence of SM’s professional background and preconceptions on the study, reflexivity was emphasised throughout data collection and analysis. SM maintained a reflexive diary to document his positionality, assumptions, and reflections on how his clinical experience might shape interactions and interpretations. Regular discussions with the broader research team ensured that multiple perspectives informed the analysis, strengthening the trustworthiness of the findings.

### Observation shifts

SM conducted non-participant ethnographic observations [19] from July to October, 2022 in the LAS/LAA Emergency Operations Centre, focusing on the use of video livestreaming for decision-making. Data were anonymised and collected through field notes and study-specific data-collection proformas.

### Semi-structured interviews

#### Participant selection

Clinician dispatchers were invited via email to participate in semi-structured interviews. The purposive sample included a range of ages, genders, job roles, and experiences with video livestreaming. The interviews were undertaken during the same time period (July to October 2022) as the observation shifts but were conducted separately, at times when participants were not actively on shift.

#### Data collection

Interviews, conducted by SM via Microsoft Teams™, lasted between 45 and 90 min. An iteratively developed interview topic guide drew on NDM theories [17, 20]. Interviews were audio-recorded, transcribed verbatim, and analysed without participant review of transcripts.

#### Analysis

Thematic analysis using the framework method [21] was employed to analyse interview and observation data. Inductive and deductive coding identified themes based on NDM [16] and SA theories [17]. The qualitative subgroup developed an analytical framework and applied it to all data. Observational and interview data were triangulated using the framework method to enhance the validity of the findings. The analytical framework was applied to all data, with NVivo software [22] assisting in coding and framework matrix creation.

## Results and findings

Approximately 200 h of observation were completed during routine shifts (including day and night shifts and different days of the week). The observation periods ranged from 4 to 12 h in duration. Video livestreaming was used 39 times (ranging from 0 to 5 in individual observation periods).

The study involved 17 participants in total; 15 of whom were observed in practice and 13 participated in interviews. Our sample included 13 males and 4 females, with 10 LAA paramedics and 7 APP-CC. Participants'experience in their current roles ranged from 0–4 years (*N* = 9) to 5–9 years (*N* = 8). Most participants (14/17) had 3 years of experience using livestreaming, while two had 1 year and one had 2 years.

### Findings

**Fig. 2:**
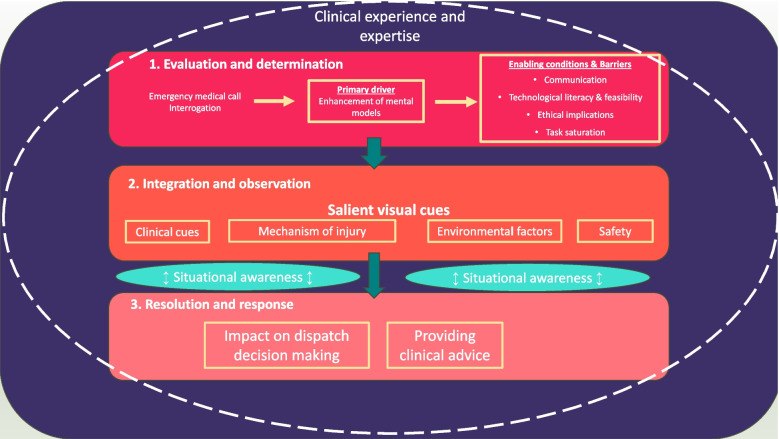
Conceptual model of the use of video livestreaming in trauma dispatch

Following thematic analysis, three distinct phases were delineated for how video livestreaming influenced the dispatch decision-making process (see Fig. 2). These phases and associated themes, along with an additional overarching theme (Interweaving expertise and technology in decision-making), were used to structure the findings presented below. Each phase addresses specific aspects of the research question:

- Phase 1 Evaluation and determination explores why video livestreaming is used and the patient/caller, incident and participant characteristics, focusing on the initial assessment and feasibility of video livestreaming during emergency dispatch.
- Phase 2 Integration and observation: examines how livestreaming impacts situational awareness, detailing the visual cues that aid the participants.
- Phase 3 Resolution and response: addresses the'impact'of video livestreaming on dispatch decisions and immediate care advice provided to callers.

The overarching theme, Interweaving expertise and technology in decision-Making, highlights the role of the participants’ clinical intuition and experience.

#### Phase 1: Evaluation and determination

The initial phase of decision-making involved the participants determining whether it might be helpful and feasible to use video livestreaming and included the following themes: interrogation of emergency medical calls, primary drivers for using video livestreaming, and enabling conditions and barriers to its use.

Interrogation of emergency medical calls

**Fig. 3:**
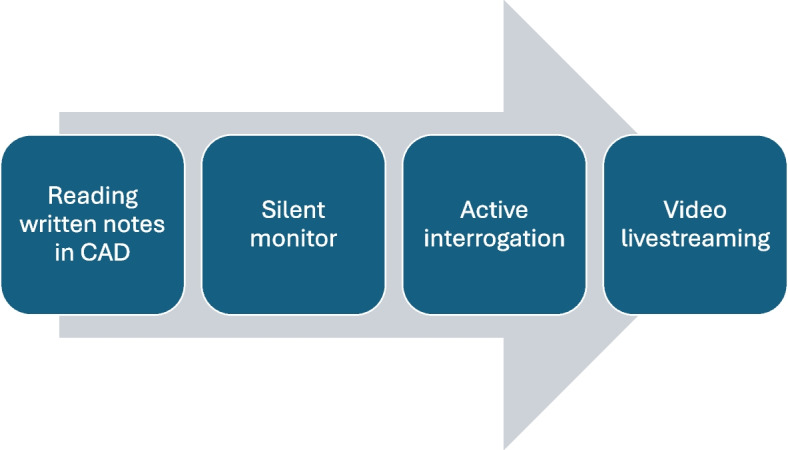
Modes of interrogating emergency medical calls. CAD = Computer Aided Dispatch

Identifying trauma incidents to send the Enhanced Care Teams was described as challenging owing to the high volume of calls coming into the EMS system each day and having to piece together incomplete information. The first process the participants would undertake to obtain information was a multi-tiered approach they described as ‘interrogating’ the incident (see Fig. 3). This process describes how participants gather and assess information to determine the necessity of video livestreaming.

One participant described the process leading up to the decision to use video livestreaming at interview:

*“It’s where you don’t have enough information and you cannot picture the call and normally it starts with, if there is a hierarchy—you read the [computer aided dispatch notes]—not enough information; listen in—not enough information; then you would do active interrogation… and then if you think after the active interrogation I still don’t have enough information then I would move to video streaming, so I would do a tiered approach to it.”* (P11)

This participant described a tiered approach to deciding on the use of video livestreaming. However, observations revealed that the process appeared more flexible than strictly hierarchical. Other participants were observed to often bypass earlier stages, opting directly for video livestreaming after reviewing the Computer Aided Dispatch notes. Furthermore, participants had the discretion to exit the process at any point, proceeding with action once they had gathered enough information to decide.

The LAA participants also had formal dispatch criteria that would elicit an immediate decision to dispatch the HEMS team. While there was an expectation that the HEMS team would be dispatched immediately if the emergency medical calls met the criteria, these incidents were also interrogated, and participants would use their discretion and experience to decide whether to rigidly adhere to the criteria.

##### Primary driver: enhancement of mental models

When there was insufficient information to gain an accurate mental model (described as being ‘on the fence’), video livestreaming was considered a useful tool. This explains why video livestreaming is used – to enhance participants' mental models when information is incomplete, which we have labelled the ‘zone of uncertainty’ (see discussion), and how video livestreaming aids in decision-making in these situations.

*“And actually, I think that [video livestreaming] is for the on the fence kind of injuries, not the blindingly obvious…I think, actually, I’m more [video livestreaming] the ones where I have been on the fence.”* (P03)

A mismatch of information was frequently noted during observation shifts and reported by the participants between what the emergency medical callers described on the phone and what was seen and understood by the participants once video livestreaming was activated. For example, during one observation period, a caller reported that a heavy object, estimated by the caller to be 200 kg, had fallen onto a patient's leg, trapping them, and causing severe bleeding from a wound. Using video livestreaming, the participant observed that the patient was not trapped and had only suffered a minor leg wound that was not bleeding. Consequently, the participant advised the patient to visit the hospital accompanied by a friend, avoiding the need for Enhanced Care Team dispatch.

##### Enabling conditions and barriers

Enabling conditions and barriers, while not serving as direct catalysts for the initiation of video livestreaming, play a crucial role in its efficacious use. These factors included communication challenges; technological literacy and feasibility; ethical implications; and cognitive load.

##### Communication issues

Effective communication with callers during video livestreaming was considered crucial for its success. Factors affecting communication included the caller's comprehension, state of mind, suspected intoxication, language barriers, and environmental noise.

##### Technological literacy & feasibility

The caller's familiarity with technology and the feasibility of using video livestreaming, such as smartphone availability and signal strength, were critical considerations. Participant 03 identified callers being unable to initiate the video livestreaming function due to not owning a smartphone or lacking the technological skills, as significant barriers:

*“I think the sort of barrier there is… is… is the person using the technology. Either they don’t have a smartphone, or… or they can’t use it. And I think that’s… that’s the barrier to the… to the… to being able to use it…Or someone that just doesn’t understand, you know, technology and smartphones.”* (P03)

##### Ethical implications

The participants described several ethical issues they considered when deciding to use video livestreaming, for example, instances where the patient is unable to consent (e.g. unconscious); where they believe it would not be dignified for the patient; or where the caller or participant may be at risk of danger from using video livestreaming, either physically (caller) or psychologically (caller or participant). Participant 02 reflected on the delicate balance between beneficence and non-maleficence in video livestreaming:

*“If I think [video livestreaming] is going to aid my assessment whilst still maintaining their dignity, and my professionalism as a registrant…then I will instigate that. But you’ve got to make all of those decisions really quickly. So, you’ve got be clear in your head that what you’re doing is the right thing for the patient at that time, as their advocate, without compromising their privacy, their dignity.”* (P02)

##### Task saturation

Video live streaming was reported to demand significant cognitive resources, potentially impacting the participant’s ability to attend to other emergency medical calls and tasks effectively. One participant discussed how the focused attention required for undertaking video livestreaming on an individual emergency medical call might compromise their capacity to manage the wider situation of other incoming calls and tasks.

*“Yes, it’s distracting you from a control room set up point of view…the minute you are engaged in [video livestreaming] you are not looking at the flow and you could miss something.” *(P11)

#### Phase 2. Integration and observation

This phase addresses how participants integrate and interpret visual information from live streamed footage to make informed decisions about deploying Enhanced Care Teams.

##### Visual cues

When viewing live streamed footage from incidents, participants noted the use of visual cues in ascertaining the necessity for deploying Enhanced Care Teams. These cues, supplementing insights acquired from previous interrogation methods, enhance the participants'comprehension of the unfolding situation and the patient's requirements. The visual cues reported by the participants were categorised into four distinct types (Table 1).

**Table 1:** Salient visual cues when video livestreaming

**Cue classification**	**Examples of cue elements**
Clinical cues	Level of consciousness; wound assessment; pallor; respiratory effort; external blood loss
Mechanism of injury	Damage to vehicles; height of fall; distance ejected from vehicle; viewing the item that caused injury
Environmental factors	Access and egress information; location of incident; whether additional agencies are required for accessing/extricating patient; traffic conditions
Safety of the scene	Hazards; aggressive people on scene; weapons present; police presence

##### Clinical cues

Clinical cues included assessments of the patient’s responsiveness or level of consciousness; observations of external blood loss; open limb fractures; and the size, location, and depth of wounds, as described by one participant:

*“If there’s calls that come through and we’re sort of looking for a bit more information, be it looking for the level of responsiveness, or you know, a breathing status, or even looking at the sort of the site, or type of wound, [video livestreaming] really comes in handy for that.”* (P12)

##### Mechanism of injury

The participants look for visual information that provides greater insight into the mechanism of injury, such as damage to vehicles or the height someone has fallen. One participant described how having eyes on the scene enabled them to gather more contextual information to aid decision-making:

*“You think, ‘okay, so let’s start thinking about mechanism. I’ve got an RTC. This is a big road. I can see it’s a dual carriageway. The tank is dented. The handle’s missing. [The patient] is down the road.’ All of that, and I haven’t even spoken to [the patient]”* (P02)

##### Environmental factors

Environmental factors include understanding incident location information, such as access and egress. An observation example was a reported incident involving a patient allegedly trapped with a possible open leg fracture, in what was referred to as a ‘big hole’. Confronted with ambiguous descriptions and unable to visualise the scene, the participant used video livestreaming. This revealed that the patient was a construction worker injured within a large excavation site. Through video livestreaming, the participant ascertained that the injuries were not severe enough to warrant an Enhanced Care Team dispatch and confirmed that there was sufficient access to and from the excavation site for standard emergency response.

##### Safety

Participants use visual information from video livestreaming to assess the safety of the scene and any potential hazards or threats. Participant 12 elaborates on how participant’s leverage video livestreaming to gauge the safety and potential risks of a scene, including the presence of aggressive individuals or hazardous environments. They explain:

*“You’re looking at the general scene, so you can have a brief idea of things like, is it a hostile scene? Is it safe for the team to approach? Are Police sort of nearby…So, I’m looking at things about, you know, the tone of the scene, as I said. Is it a chaotic scene? Is there an assailant? Is the patient in danger?”* (P12)

#### Phase 3. Resolution and response

The final phase includes resolution and response derived from the use of video livestreaming in the dispatch process. This phase addresses how video livestreaming influences dispatch decision-making and the provision of clinical advice to callers for immediate care. It highlights how both participants and patients benefit from the enhanced decision-making and immediate clinical advice provided through video livestreaming.

##### Impact on dispatch decision-making

The participants considered the efficient and accurate dispatch of Enhanced Care Teams to be the main goal of their role, as these resources are limited and in high demand. The participants discussed the implications of incorrectly dispatching Enhanced Care Teams to incidents where they were not needed, leading to the reduced availability of Enhanced Care Teams for other patients that require specialised skills.

*“I guess it’s where we’re trying to find five or six patients in a 24-h period and we’re trying to find the ones that are the most sick out of say 6,000 or 7,000 calls…I guess I’m trying to work out what injuries they have, the severity of it and what the benefit from a [air ambulance] team would be for this patient.”* (P05)

Below, Participant 06 described their decision-making process as a threshold on a scale on whether to dispatch the ECT or not. As they gain more information, the incident moves up or down on the scale. Video livestreaming was described as useful for incidents that did not meet the threshold of whether to dispatch a team, but visual confirmation can influence that decision.

*“I picture it like there’s a line, there’s a line at which that threshold is met and you’re going to commit the team to that job…So, that line moves as I hear stuff, as I go through the silent interrogation, as I actively interrogate. Or as I [live stream], [the decision] moves up and down that curve.”* (P06)

##### Provision of clinical advice to callers to assist in immediate care

Participants discussed how video livestreaming has provided a new opportunity to move beyond dispatching, and provide a conduit for providing immediate clinical advice to callers, signifying what was described as a ‘culture change’ in their role:

*“…we can all dispatch, but actually, this is about starting treatment before the medical team get there because you’re stating that this person’s critically unwell, we should be starting treatment and this is what [video livestreaming] allows you to do, to be able to see and use it as your eyes to be able to provide treatment prior to the medical team. And that’s the sort of culture change with inside all of our control rooms because we’re so used to control rooms doing just about dispatch and not about treatment.”* (P01)

The participants were able to guide callers through essential first aid procedures, leveraging the visual information to provide accurate and situation-specific instructions. The interviews and observations revealed specific instances in which this technology was employed, such as haemorrhage control, wound dressing, airway management, and patient positioning.

#### Overarching theme 4: Interweaving expertise and technology

All participants highlighted the critical role of clinical intuition and judgment in the dispatch of Enhanced Care Teams. Terms such as “*Spidey sense*” (referring to spiderman) or “*gut instinct*”, were used. Participant 03 discussed how they believed this intuition was developed through the participant’s clinical experiences, to identify which patients would benefit from receiving an ECT.

*“…having a clinician in the room, I think you get a sense when you think something’s a HEMS jobs. Something that can benefit from the critical care team…I think, at the start, it’s the spide-y sense, the tingle…”* (P03)

Many participants believed their expertise enabled them to recognise and interpret the significance of the visual cues observed through video livestreaming. This expertise, honed by exposure to various emergency scenarios, allowed participants to identify patterns and assess the severity of incidents.

*“I think it all comes down to kind of your clinical experience and knowing the patients, and really knowing what you as a system can deliver and the patients that you care for, to then taking that back and looking for those patients with livestreaming in the control room”.* (P05)

## Discussion

Previous literature on video livestreaming in emergency medical dispatch has focused primarily on its use for specific interventions, such as dispatcher-assisted CPR [23–25], with limited exploration of its broader application in scenarios like trauma incidents or its impact on decision-making and situational awareness [9]. As highlighted in a recent scoping review, much of the current evidence is based on simulation studies, with few real-world evaluations addressing the acceptability, effectiveness, and challenges of video livestreaming [9]. This study uniquely contributes to the literature by triangulating observation and interview data to develop a comprehensive model that explains how video livestreaming influences situational awareness and decision-making in trauma-related emergency dispatch. The findings highlight how the utility of this technology varies depending on patient or caller characteristics, incident specifics, and clinician dispatcher attributes, offering nuanced insights into its potential benefits and limitations.

The findings from this study suggest that video livestreaming enhances all three levels of Endsley’s model of situational awareness [17] —perception of elements in the environment and comprehension of their meaning—by providing real-time visual data, which helps the participants perceive and comprehend the scene more accurately. At the third level, projection of future status, the visual information and cues supports clinician dispatchers in anticipating the likely progression of the incident and making informed decisions regarding resource allocation and immediate clinical advice. Video live streaming was found to be particularly useful for ambiguous incidents where the participants struggle to form a clear mental model based on audio information alone. Conversely, this technology is less beneficial for incidents that are immediately clear-cut, where traditional methods of interrogation suffice. These findings align with previous research on video livestreaming in EMS dispatch [8, 26, 27], which indicated that operators tend to use video selectively, primarily in situations where they are uncertain about the situation or the patient’s condition. Participants in our study expressed that such uncertainty increases the risk of both over-triage, potentially dispatching specialist teams to incidents where they are not required, making them unavailable for other critical emergencies, and under-triage, delaying the deployment of specialist resources to patients in severe conditions. Observations from this study suggest that video livestreaming may help mitigate these risks by providing additional visual data, enabling dispatchers to feel more confident in their situational analysis and decision-making regarding resource allocation and patient care.

**Fig. 4:**
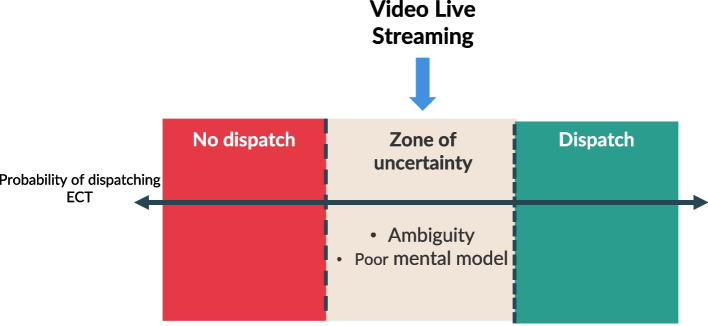
Impact of Livestreaming on decision dispatch decision making (adapted version of threshold decision making model [29])

Our study adds to this discussion by introducing the concept of the *'zone of uncertainty,'* where the clinician dispatcher’s situational awareness is unclear, and decision-making is challenging (see Fig. 4). Video livestreaming plays a crucial role in shifting decision-making across this threshold of uncertainty. Our findings extend the Threshold Decision-Making Model, which we suggest clinician dispatchers use, to include this concept. The Threshold Decision-Making Model [28, 29] is a framework employed to determine whether to take action based on the probability of a certain outcome. This model, which has been studied in other healthcare settings [28, 29], suggests that if the estimated probability of a condition or event exceeds a certain threshold, action is taken (e.g., dispatching the ECT). By introducing the idea of the zone of uncertainty, our study extends this model to the context of dispatch, demonstrating how video livestreaming helps clinician dispatchers move from a state of uncertainty to a more informed and confident decision.

The participants in our study reported that video livestreaming can require focussed attention. As attention is a limited resource [30], the intensive engagement and cognitive loading discussed by the participants required to use livestreaming may divert their attention from the wider operational context, leading to a narrowed field of attention that could overlook simultaneous demands. While similar concerns were reported in a study by Idland et al. [27], some participants also suggested that video livestreaming was more useful in busier periods, as it made communication and information collection more efficient. Previous research has highlighted the cognitive overload emergency clinician dispatchers may experience whilst trying to execute multiple demanding tasks, even before the introduction of video livestreaming, and highlighted a potential trade-off inherent in the integration of technologies in emergency dispatch systems [31, 32].

Findings from the studies discussed, along with our own research, suggest that integrating video livestreaming necessitates new strategies to ensure that necessary wider system awareness can be maintained. However, the specific strategies to achieve this balance remain to be clearly defined. Further research is needed to explore and develop effective approaches that can mitigate the cognitive load on clinician dispatchers while leveraging the benefits of video livestreaming. Possible areas of investigation could include enhanced training programs, the use of Artificial Intelligence and automation, and dynamic workload management systems operations effectively.

Our study found the participant’s clinical expertise and intuition were considered to play an important role in effectively incorporating video livestreaming into the decision-making process. Their practical experience with similar patients was believed to allow them to identify which would benefit from enhanced care teams (projection of future status in Endsley’s model[17], based on their previous (or ‘prototypical’) encounters with such patients in real-world settings). This aligns with Klein’s Recognition Primed Decision Model theory [33], which emphasises the importance of experience to allow pattern recognition to make rapid, effective decisions in high-stake environments.

The findings suggest that video livestreaming's effectiveness is contingent on clinician dispatchers'ability to interpret the visual information from complex scenarios—it is unknown how video livestreaming may impact dispatch decision-making and situational awareness in systems that use non-clinically trained dispatchers [5]. One study investigating the use of video livestreaming in a non-clinical HEMS dispatch model reported that it was feasible to implement video livestreaming in their system, however there was no investigation in to how this may have influenced or affected the dispatch decision-making process [4]. Thus, EMS systems must weigh the wider introduction of new technologies against the experience and expertise of their users, ensuring their application in justified situations and supporting their integration with adequate training.

One of the key findings from our study is that enhanced situational awareness through video livestreaming enables participants to provide immediate clinical care remotely to patients. This can only work in systems with clinically trained operators and within governance systems that allow such intervention. Previous literature has shown that video livestreaming can have utility for remote intervention in the context of cardiac arrest with clinically trained operators [9]. This capability moves beyond purely dispatching and positions clinician dispatchers as conduits for real-time medical assistance. Idland et al. [34] conducted a study investigating the association between video streaming and the recognition of first aid needs during emergency calls. Their findings suggest that video livestreaming is associated with improved recognition of patients’ first aid needs and can aid dispatchers in guiding bystanders more effectively. However, they did not find a significant association between video streaming and the overall quality of bystander first aid.

Our research indicates that caller characteristics influence the effectiveness of video livestreaming. This finding is consistent with that of Linderoth et al. [8], who also reported that callers in emotional distress, having language barriers, and difficulty in following instructions posed challenges for clinician dispatchers using video livestreaming. However, these findings are not specific to video livestreaming for emergency medical calls, as Higgens et al. [35] reported similar challenges during audio-only emergency medical calls. The consistency of these findings across both video and audio calls suggests that the effectiveness of emergency dispatch is fundamentally influenced by the ability of the emergency medical caller and clinician dispatcher to communicate with each other effectively. Despite the addition of visual information, clinician dispatchers still need to have excellent communication skills and be adept at calming distressed callers, providing clear and concise instructions, and adapting their communication style to suit the caller's needs and technological capabilities.

Previous literature has highlighted the limited exploration of the ethical aspects of video livestreaming in EMS, particularly regarding caller and patient consent process, privacy, dignity and the potential psychological impact on caller and clinician dispatcher [9]. Our study adds to the literature by reporting how clinician dispatchers actively navigate ethical challenges when using video livestreaming, including balancing the need for improved situational awareness with concerns about patient privacy and dignity, especially in cases where patients cannot provide consent. Participants emphasised the importance of acting in the patient’s best interest while adhering to professional ethical principles. Despite these insights, further research is needed to address persistent gaps, such as establishing robust governance frameworks, refining consent processes and understanding the broader implications of video livestreaming on caller and patient autonomy.

### Strengths and limitations

This study's strengths include its multi-method qualitative approach and real-world data collection, providing valuable insights into how video livestreaming is perceived to influence EMS decision-making. However, the findings may not be fully transferable to rural settings or EMS systems with non-clinical dispatchers. Additionally, ambulance resources, skill levels, system modelling, and dispatch criteria vary widely across the world, which may impact the transferability of our findings to different EMS contexts. Another limitation is that this study did not have access to the total number of calls to the emergency operations centre during the study period, which limits the ability to compare how often livestreaming was used in the context of all incoming calls. Additionally, the study focused solely on trauma incidents and its findings may not be transferable to medical or non-trauma incidents, or lower acuity cases.

Further research is needed to explore the effectiveness of video livestreaming across diverse EMS systems, including rural areas and systems with varying levels of dispatcher expertise and resource availability. The rapid international uptake of this technology [9] underscores the importance of understanding its optimal application. Future research should examine not only its use in a broader range of incident types but also the psychological impact on dispatchers and callers, strategies to address these challenges, and the ethical considerations surrounding its use. Importantly, video livestreaming is a complex intervention within a highly complex system, warranting further research focussing on identifying the mechanisms through which video livestreaming delivers benefits, the contexts in which it is most effective, and the specific circumstances and users for whom it works best [36].

## Conclusion

This study demonstrates that video livestreaming can impact decision-making in emergency dispatch for certain incidents and patients, especially in complex trauma scenarios. Understanding and addressing factors influencing its effectiveness can help EMS systems maximise its benefits, potentially improving patient outcomes and resource allocation. However, further research is needed to explore its impact in rural areas and systems that use non-clinically trained dispatchers Table 2.

**Table 2:** Glossary of terms

APP-CC	Advanced Paramedic Practitioner – Critical Care	A specialist paramedic affiliated with the London Ambulance Service NHS Trust, equipped with post-graduate education and expertise in prehospital critical care. Their responsibilities include direct patient care and operating the Advanced Paramedic Practitioner Critical Care desk, where they oversee the deployment of other APP-CCs and offer guidance to ambulance teams remotely
CAD	Computer Aided Dispatch System	A software system used by Ambulance Trusts for evaluating emergency calls and coordinating the deployment of ambulance services
ECT	Enhanced Care Team	A term encompassing both Advanced Paramedic Practitioner – Critical Care (APP-CC) and London's Air Ambulance
EMS	Emergency Medical Service	
EOC	Emergency Operations Centre	A facility dedicated to receiving and prioritising emergency medical calls from the public and other emergency agencies such as police, fire services, and coastguard. It plays a crucial role in orchestrating the response and allocation of resources to various incident locations
HEMS	Helicopter Emergency Medical Service	A service providing immediate prehospital emergency and critical care to patients, using helicopters and/or road vehicles. The teams are composed of doctors and paramedics, trained to handle critical situations
LAA	London Air Ambulance	A charity that delivers an advanced trauma team to critically injured patients in London with a doctor and paramedic team. Operating via helicopter during the day and rapid response cars at night or in poor weather
LAS	London Ambulance Service NHS Trust	London Ambulance Service NHS Trust

## Supplementary Information

Supplementary Material 1.

## Data Availability

The datasets used and/or analysed during the current study are available from the corresponding author on reasonable request.
