# Supplementary material for: Video livestreaming in emergency trauma dispatch: an observational study of technological integration with clinical decision-making in prehospital enhanced care services
Source: Scand J Trauma Resusc Emerg Med. 2025 Jun 19;33:108. doi: 10.1186/s13049-025-01406-2 (PMC12177982; doi:10.1186/s13049-025-01406-2)
Supplement: Supplementary file 1 — Supplementary Material 1. [file 13049_2025_1406_MOESM1_ESM.pdf]

Emergency Medical Services Streaming Enabled Evaluation In Trauma: The SEE-IT Trial.

Topic guide: LAS/LAA dispatchers

Introduction

Thank you for volunteering to participate in this interview. As you are aware, researchers at the University of Surrey are testing live video streaming for trauma dispatch.

The aim of our research is to see if using a smartphone camera and live streaming from the scene helps you to make quicker and more accurate decisions about the help that is required. I want to understand if using live streaming is acceptable to dispatch control room staff. As you may be aware, the focus of this study is on 999 calls involving trauma.

As outlined in the accompanying information, I would like to speak to you about your experiences and I would like to record our conversation today with your permission. Are you happy for me to record our interview? I may wish to use anonymised extracts or quotes from this interview when we produce summaries of our findings. Are you happy for us to do that?

[If yes, START RECORDING]

Please could you confirm for me if you agree to participate in this study and do you feel comfortable talking about your experience of using live video streaming for dispatching to trauma incidents?

Please let me know if you feel upset and we can pause or stop at any time. If you do not feel comfortable answering a question, please tell me and we can move on.

Opening questions

Q: Please can you tell me about your role and what your responsibilities are? How long have you been in your role?

Receiving 999 calls

Q: I would like to start off by asking you to describe what *normally* when you respond to a 999 trauma call in the EOC?

Probes:

- What protocols do you draw on/refer to?
- In what way do they help you make dispatch decisions?
- In what way do you draw on your experience, knowledge, and professional training to interpret the situation and take appropriate action?

- What role does intuition play?
- Describe how you draw on the caller's description of the scene to make decisions. What information do you ask the caller for, what helps you most, and what helps you least about the information they offer? What are the cues?
- How reliable is the information passed by the callers?

- Could you describe how the final decision about dispatch is made?

Q: Are there things that make it harder and/or easier to initiate an accurate and timely emergency response for trauma calls?

Probes:
- Describe what makes the accurate response *more difficult* - these could be related to organisational, financial, team-working, personal attributes, leadership during shifts training, or resource issues for example.
- Describe what makes accurate response *easier* - these could be related to organisational, financial, team-working, personal attributes, leadership during shifts training, or resource issues for example.

Questions regarding live streaming

Q: how many times approximately have you used GoodSAM for trauma calls? (How long have you been using it?).
- Would you say you use it often/not often?
- Are you able to estimate how often?

Questions on training, procedures

Q: Can you tell me about the training you received to use GoodSAM?
- Was there enough information to help you use the system?
- Was there a formal training package (and who was it delivered by) or was it on-the-job training?
- Was there a formal sign-off?
- How did you demonstrate you were competent to use it once trained? How long did it take you to feel confident using it?

Q: Are there formal organisational procedures/guidance on the use of live video streaming?

Q: Describe the process of consenting/asking the 999 callers to use the link for GoodSAM: Is there a script that you use?

Q: How useful is it to be able to live stream? Does it make your job easier or harder? How does it affect the rest of your work as a dispatcher?

Questions on diversity

I would like to know more about how patients' characteristics and their diversity play a role in the use of video streaming. I'm going to ask you some questions about age, gender, ethnicity, and language if you could think about how these influence your or their choice on whether to use video streaming, how they influence how effective it is

or what might happen during a video stream, whether particular aspects of these characteristics make it easier or more challenging to use.

Q: Can you tell me about how the caller's (or patient's) language may influence your decision-making to use live streaming or its effectiveness when using it?

Q: Have you had experiences where the patient's culture or religion has affected the use of video streaming? If yes, can you tell me about it? What particular aspects?

Q: How does the caller or patient's age influence your decision-making or ability to use live streaming or how effective live streaming has been? Can you give me examples of incidents where age might have been an influence? (e.g., consenting a child, asking an elder patient to use the technology)

Q: Are there any particular patient groups that respond better or worse to using live streaming?

Q: Can you tell me how easy or difficult you find using live streaming while working as a dispatcher?

Q: How do you decide which jobs to use live streaming on?

- Are there particular indicators that would make you want to use it? E.g., type of job, lots of calls coming in about the same job, 'red' flags.
    - Are there any jobs that have surprised you, where you did not think GoodSAM would be that helpful, but it was?

- Is there anything that would make you decide not to use it? Sounds like a stressful/dangerous scene; caller too young; concern over patient dignity; concern over scenes being too distressing to view

Q: What type of information are you trying to gain by seeing the live stream of the scene? E.g., Injury, scene, the status of the patient, safety, etc.

Q: How does having the visual information of the scene affect your ability to accurately dispatch, compared to just verbal communication over the phone?

Q: When using live streaming, are you able to communicate with the caller in a way that you normally do without live streaming? What is different?

Q: Does the streaming hinder effective and accurate dispatch in any way?

Q: Can you think of any examples where you did not use live streaming, but wish you had in retrospect?

Q: What is your experience with the reliability and use of the technology?
- quality of interface (picture and audio/sound), problems with the signal, and people not having smartphones.
- Are there any common technical problems you encounter?

Q: How do callers react to being asked to use live streaming? Do most agree/disagree to help with this?

Q: What factors make people unwilling or unable to use live streaming when requested?

Q: How do other people on scene react to the callers using their phones to live stream? Does it seem to be well/poorly received? Do you think the other people understand what is going on? If there are common problems, how do you navigate them?

Q: Are there any common problems or complications you or the callers find when trying to use live streaming? (They call from a landline; the scene is too dark; people unable to follow instructions)

Well-being

Q: Do you think live streaming could cause risks of harm to the caller/patient/dispatcher? (Ask as separate questions)
- Are you able to think of an example? Have you seen any evidence of harm to a 999 caller by using live streaming?

Q: Do you think the use of live video streaming has an impact on the rest of the EOC staff around you? Is there a risk it could also harm them?

Q: What impact does seeing the streamed footage from the scene have on you?

- Does viewing the live-streamed footage ever cause you distress? If yes, was it at the time or after the event? Can you offer any reasons as to why you think this?

Concluding questions

Q: Are there any questions that we have not asked you that you were expecting? Why do you think this is important to ask about?
Q: If I have any further questions from today, would you mind if I contacted you by telephone or email to clarify?
Q: Would you like a copy of the final report when the study finishes? If so, how should we send this to you? (email/postal address and give them an indication of when this might be).
